# Goji Leaves and Buds as Food Resources: Composition, Processing, Safety, and Links to Side-Stream Valorization

**DOI:** 10.3390/foods15183298

**Published:** 2026-09-17

**Authors:** Jinhao Zou, Xiaowei Tian, Siyi Wang, Ye Sun

**Affiliations:** 1 College of Pharmacy, Ningxia Medical University, Yinchuan 750004, China; pullenda20@gmail.com; 2 Key Laboratory of Dryness Syndrome in Chinese Medicine, Ministry of Education, Ningxia Medical University, Yinchuan 750004, China; 3 College of Traditional Chinese Medicine, Ningxia Medical University, Yinchuan 750004, China

**Keywords:** *Lycium barbarum*, *Lycium chinense*, goji leaves, bud tea, food processing, food safety, by-product valorization, circular food systems

## Abstract

Goji leaves and buds are used as vegetables and teas, but their composition and behavior in formulated foods differ from those of the berries. This critical narrative review examines their phytochemistry, processing, food applications, digestion, and safety, with selected comparisons to berry-processing residues and orchard biomass. The literature includes leaf-tea processing, polysaccharide-modified dough, emulsion delivery systems, and bud-tea or leaf-extract additions to savory foods. Genotype, harvest stage, and extraction account for substantial compositional variation. In food matrices, improvements in rheology, oxidative stability, or flavor depend on formulation and dose—higher inclusion does not consistently improve sensory quality. Simulated digestion and biological models provide preparation-specific findings but do not establish clinical efficacy. Safety evidence includes a short-term toxicology study of one roasted *Lycium chinense* leaf extract and pesticide occurrence and infusion-transfer measurements in bud tea. Neither dataset establishes safety across all leaf-derived products. Berry residues have separate applications in bakery products, ingredient recovery, and animal feed, whereas woody branches are predominantly agricultural biomass. Extraction yield alone establishes neither food suitability nor environmental benefit. The main unresolved issue is whether the compositional and technological effects observed in individual experiments persist across production batches, realistic servings, storage, and longer-term exposure.

## 1. Introduction

Goji berries from *Lycium barbarum* L. and *Lycium chinense* Mill. have received considerable attention as foods and sources of phytochemicals [1,2,3,4]. Leaves and buds have a different food history and chemical profile. They can be harvested as vegetables or processed into tea, whereas mature woody branches and berry pomace originate from orchard management and fruit processing, respectively [5]. These materials differ not only in plant organ but also in the fraction consumed: fresh leaves, an infusion, a purified extract, and a seed-rich pressing residue expose consumers to different mixtures.

Food research on goji leaves has expanded since the leaf-focused review published in 2022 [5]. Studies now compare tea aroma across plant lines, distinguish bud from mature-leaf tea, and examine fermentation, polysaccharide-modified dough, colloidal delivery systems, and savory-food formulations [6,7,8,9,10,11,12]. Pesticide-transfer measurements in bud tea also permit closer examination of the relationship between the dry ingredient and the consumed infusion [13]. Together, these studies raise a more specific question than whether leaves contain bioactive compounds: how do their constituents behave during processing and in the foods in which they are used?

Existing reviews cover whole-plant utilization, bioactive constituents from different organs, flavonoid processing, seed products, and pharmacological applications [14,15,16,17,18]. The present review focuses instead on leaves and buds as food resources, particularly the relationship between composition, processing, matrix performance, digestion, and preparation-specific safety. Berry-processing residues are considered where food incorporation or ingredient recovery provides a relevant comparison, while feed and orchard-biomass routes receive a shorter, separate treatment. Detailed root-bark pharmacology and comprehensive seed chemistry are outside this scope. Supplementary Table S1 summarizes the coverage of related reviews.

The distinction between harvested foods and residual biomass also affects the interpretation of valorization. Deliberately produced leaf tea is not a fruit-processing by-product. Conversely, pomace used in bakery products, peptide discovery, volatile release, or oil recovery can contain different proportions of pulp, peel, and seed [19,20,21,22]. Treating these fractions as interchangeable obscures both their technological properties and the additional processing required for their use.

## 2. Review Approach

### 2.1. Search and Selection

This critical narrative review used the archived PubMed and Europe PMC searches updated to 29 July 2026, supplemented by OpenAlex, Crossref, publisher searches, and citation checking. Queries combined *Lycium*/goji/wolfberry with leaf, bud, shoot, and defined residue terms and with food-processing, composition, digestion, safety, feed, and biomass terms. A targeted update during revision on 6 September 2026 examined additional recent processing and agronomic records. Web of Science, Scopus, FSTA, and CAB Abstracts were not searched, so food formulation, cultivation, feed, and industrial-processing studies outside biomedical indexes may, therefore, be underrepresented. The relative amount of evidence discussed for each material cannot be interpreted as the relative extent of research on that material. Exact archived queries, source dates, and the separately logged revision update are provided in Supplementary Methods S1 and Data S1a and S1b.

Primary studies were retained when the tested material could be assigned to goji leaves or buds, or to a defined comparator organ or side stream. Species identity was retained as reported, chiefly *L. barbarum* or *L. chinense*. Commercial products and abstract-only records without an assignable species were retained as source-defined materials, not treated as species-confirmed observations. Reviews supplied context, and official documents supported jurisdiction-specific regulatory statements. Title grouping and DOI reconciliation were performed with a project-specific Python script (version 3.12.14 for the final consistency check) using normalized titles and DOI strings—conflicting title–DOI associations were not automatically merged. The archived discovery counts describe retrieved records, not a prospectively screened set of included studies. No systematic review completeness claim or quantitative pooling is made.

### 2.2. Source Access and Evidence Description

For each cited source, the register records material identity, preparation, test system, endpoint, and source-access status. Full text, abstract-level information, and official documents are distinguished in Supplementary Table S2 and the application tables. Detailed composition values were retained only where the full text and reporting basis could be checked (Supplementary Table S3). Missing information is distinguished from fields not applicable to contextual or regulatory sources. The archived ClinicalTrials.gov check used four exact leaf-related intervention terms—it was not a comprehensive search of human studies, synonyms, buds, or residues.

Evidence domains are described without scores or ordinal development stages: composition, processing or formulation, food-matrix performance, simulated digestion or fermentation, biological models, toxicology or exposure, and target-use studies. These domains address different questions and can coexist in one paper. Interpretation considers the tested species, preparation, dose, comparator, and outcome—it does not combine separate preparations into a single validated product. A formal design-specific risk-of-bias assessment was not conducted. AI-assisted preparation is disclosed in the Acknowledgments.

## 3. Botanical Identity and Uses of Leaves and Buds

### 3.1. Species, Cultivars, and Harvested Tissues

*L. barbarum* and *L. chinense* belong to the Solanaceae, but leaf chemistry cannot be assumed to be identical between them. Within *L. barbarum*, a direct comparison of fruit, leaf, and root-bark extracts found organ-dependent constituent profiles [23]. Leaf production is also an agronomic objective in its own right: planting density and cutting height experiments in *L. chinense* examined harvestable foliage rather than incidental orchard waste [24]. A recent *L. chinense* breeding study described the tetraploid cultivar Cheongsoon, selected for sprout and leaf production, with broader, thicker leaves and greater sprouting capacity than its diploid comparator [25]. These observations connect the food raw material to cultivar selection and repeated shoot harvest. Harvesting studies in *L. barbarum* further show that flowers, leaves, and fruits respond differently to vibration, affecting the composition of mechanically collected material [26].

### 3.2. Food Use and the Medicinal Root-Bark Context

The leaf review by Lei et al. describes the traditional name Tianjingcao and consumption of tender *Lycium* leaves as vegetables or herbal tea [5]. The historical account is based partly on secondary citations, whereas contemporary food experiments identify bud tea, mature-leaf tea, leaf powder, and isolated fractions as distinct preparations. Lycii Radicis Cortex, also called Cortex Lycii or Digupi, is the medicinal root bark [27]. Its pharmacopoeial context differs from that of edible leaves, and the implementation notice for the 2025 Chinese Pharmacopoeia identifies the applicable edition but does not reproduce the root-bark monograph [28].

### 3.3. Distinct Side Streams

Orchard pruning produces lignified branches, sometimes mixed with residual foliage. A dairy cow study tested branch material as a roughage replacement, whereas a sheep study used branches and leaves together [29,30]. The mixed material is neither a pure branch ingredient nor edible tender shoots. *L. barbarum* branches have also been tested as a tomato cultivation substrate, a use of woody biomass rather than a food ingredient [31].

**Figure 1:**
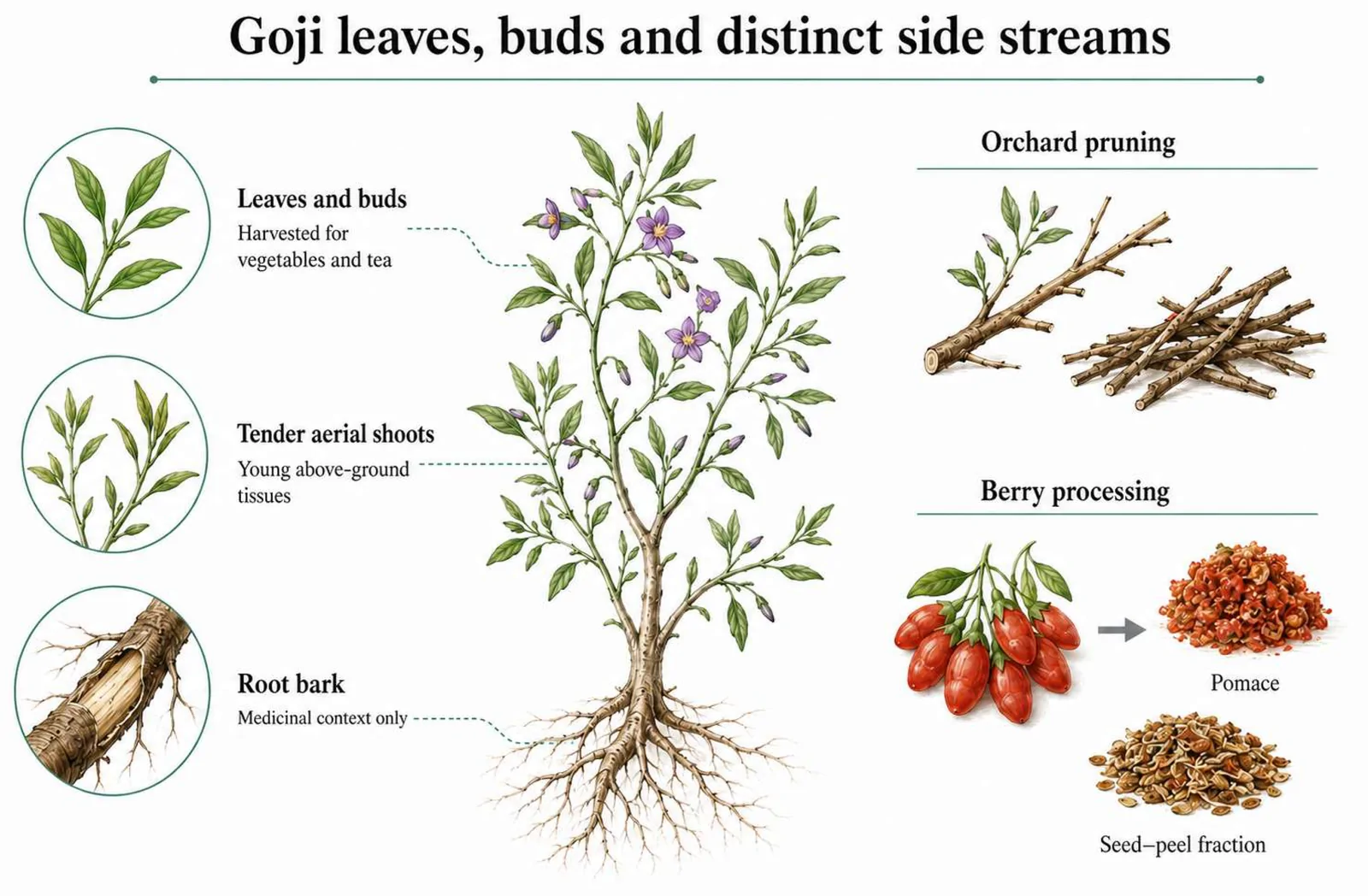
Botanical sources and production streams considered in the review. Leaves, buds, and tender shoots are food-oriented harvested tissues, and woody branches arise from pruning, whereas pomace and seed–peel fractions arise from berry processing. Root bark is shown only as medicinal context. The botanical artwork is schematic and AI-assisted, not a specimen photograph or a species identification key.

Berry-processing residues are defined by the preceding operation. Pressing, extraction, separation of seeds and peel, and fermentation produce fractions with different fiber, lipid, and soluble-component contents [20,21,22,32]. Seeds are considered here only as constituents of a defined processing residue, rather than as a separate survey of seed phytochemistry [17]. Figure 1 distinguishes these source materials from deliberately harvested leaves and buds.

## 4. Composition of Leaves and Buds

### 4.1. Leaf Nutrients, Taste Compounds, and Volatiles

Seasonal analyses of *L. chinense* leaves reported variation in micronutrients, fiber, and rutin [33], while a separate study, available here at abstract level, described seasonal sugars, organic acids, free amino acids, and volatiles [34]. These observations link harvest time to nutritional and flavor-related composition, although the abstract-only record permits less detailed appraisal. An abstract-level report of elemental analysis in *L. barbarum* leaves provides a complementary description of inorganic constituents [35]. Whole-leaf measurements and infusion composition are different quantities: brewing extracts only part of the dry material, whereas incorporation of leaf powder retains the insoluble fraction.

Genotype and tea processing modify aroma alongside these nutritional differences. Green- and white-tea procedures applied to leaves from 25 *L. barbarum* lines produced distinct volatile profiles [6]. A comparison of bud tea and mature-leaf tea also found differences in composition and enzyme inhibition responses [7]. Thus, a difference attributed to “goji leaf tea” can originate in the harvested tissue, the plant line, or the processing treatment. Within-study comparisons are particularly informative because they hold some of these factors constant.

### 4.2. Phenolics, Flavonoids, and Phenolic Amides

Rutin, chlorogenic-acid-related compounds, caffeoylquinic acids, and other flavonoid glycosides recur in leaf analyses [36,37,38]. Their concentrations differ with plant population, position on the shoot, and harvest date. Huang et al. applied reflux, ultrasound, and enzyme-assisted extraction to each of three *L. barbarum* tissues: bud, young, and old leaves [37]. The nine tissue–method combinations permit comparisons among tissues within the same extraction method and among methods within the same tissue. Zhang et al., in contrast, used HPLC fingerprints and four markers to follow harvest period differences in a fruitless cultivar [38]. Tissue maturity, extraction procedure, and seasonal variation, therefore, represent distinct sources of compositional heterogeneity. Analytical selectivity is another source of variation. In a seasonal *L. chinense* study, spectrophotometry gave much higher apparent rutin contents than HPLC on the same leaf extracts, and the authors attributed this difference to co-extracted interfering substances [33]. Such method effects cannot be explained by harvest or genotype and make total colorimetric responses unsuitable substitutes for compound-specific concentrations.

The magnitude of the reported differences is illustrated in Table 1. Dong et al. reported rutin concentrations of 16.03–16.33 mg/g dry leaf in four cultivated varieties supplied by a Ningxia institute, compared with 6.24–7.55 mg/g in wild populations from Qinghai, Xinjiang, Ningxia, and Inner Mongolia [36]. All were young leaves collected before flowering. In Romanian leaves extracted with 70% methanol, chlorogenic acid was 0.511 mg/g in a wild population and 17.811 and 24.887 mg/g in Biglifeberry and Erma, respectively [39]. Across 10 harvests of a fruitless cultivar, catechin ranged from 2.63 to 16.33 mg/g, approximately a 6.2-fold difference, while epicatechin, rutin, and chlorogenic acid each varied by more than 10-fold [38]. These are within-study contrasts, not interchangeable concentration ranges for commercial goji leaves.

Isolation studies extend the leaf profile beyond the commonly quantified phenolics, reporting sesquiterpenoid glycosides, water-soluble constituents, and compounds isolated from tender leaves [40,41,42]. Hydroxycinnamic acid amides show organ-dependent distributions in direct root-bark and leaf analyses [43]. Caffeoyl spermidine derivatives have also been recovered from *L. barbarum* leaf tea, linking this compound class to a processed beverage material [44]. A separate stem–fruit comparison illustrates further organ variation but is not evidence for the nutritional composition of edible leaves [45].

### 4.3. Polysaccharides and Other Macromolecular Fractions

Leaf polysaccharides differ with the extraction sequence and subsequent degradation. Isolation and sequential-extraction studies describe fractions with different molecular and monosaccharide characteristics, together with splenocyte or in vitro fermentation responses [46,47]. Ascorbic acid–hydrogen peroxide treatment reduces the molecular size of a crude leaf preparation and alters its physicochemical behavior [48]. Because this treatment can change more than chain length, the resulting fraction is not simply a lower-molecular-weight version of an otherwise unchanged food ingredient.

The 2021 dough study and the 2022 characterization study report crude and degraded fractions of approximately 223.5 and 64.3 kDa [48,49], whereas the 2024 dough study reports 223.5 and 94.3 kDa [9]. Their preparation histories differ. The 2021 and 2022 studies used leaves collected in August 2019 and August 2020, respectively, extracted them at 121 or 120 °C for 1 h, and selected a 0.1 M NaCl fraction by DEAE chromatography [48,49]. The 2024 study instead describes two 4 h extractions at 70 °C and AB-8 resin purification, without reporting the same DEAE selection [9]. Although all three used hydrogen peroxide/ascorbic acid degradation, the 2024 method gives a 1:1 ratio without the absolute reagent quantities reported in the earlier protocols. The preparations consequently cannot be treated as a common polysaccharide fraction varied only in molecular mass.

Analytical comparability is also incomplete. The 2022 study estimated molecular mass by refractive-index detection with dextran calibration, while multi-angle light scattering supplied the radius of gyration [48]. The 2021 report describes laser-light-scattering gel chromatography, and the 2024 report cites earlier gel permeation methods without restating the full measurement conditions [9,49]. Within the 2022 experiment, increasing degradation reagent concentration produced fractions of 187.7, 144.9, 96.26, and 64.3 kDa [48]. That within-protocol series is more interpretable than a cross-paper comparison. The difference between 64.3 and 94.3 kDa does not by itself establish a reporting error, and the documented method differences do not prove its cause. The source-specific protocols and unresolved measurement details are compared in Supplementary Methods S1.

### 4.4. Other Organs and Residual Fractions

An ethanolic *L. barbarum* flower extract contained phenolics and showed chemical antioxidant responses and mild activity against the tested Gram-positive bacteria [50]. In contrast, the stem phenolic-amide study evaluated glioma stem cell responses, not antimicrobial activity [51]. Analyses of the vitamin C analog 2-O-β-D-glucopyranosyl-L-ascorbic acid distinguish underground rhizomes from aerial stems and leaves [52]. Biogenic-amine profiles also differed between young leaves, mature leaves, and main-stem bark [53]. These organ-specific investigations broaden the known chemical distribution, although food formulation evidence remains concentrated in leaves and buds.

**Figure 2:**
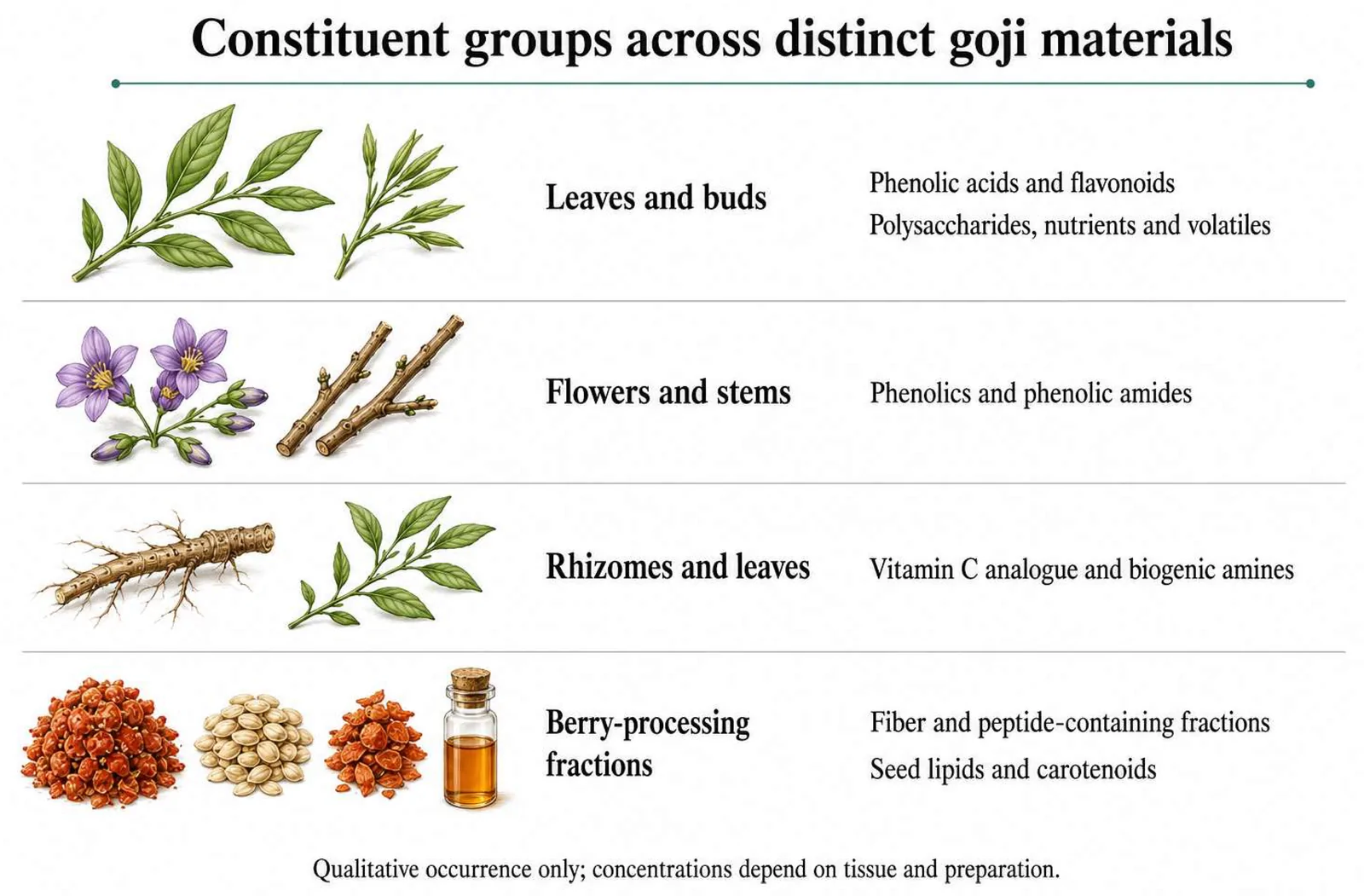
Principal constituent groups reported for goji leaves, buds, and selected comparator materials. The map is qualitative—it does not compare concentrations or nutritional value. Tender aerial shoots and underground rhizomes are distinct tissues. Illustrations are AI-assisted schematic artwork.

Berry residues provide contrasting ingredient sources. An ACE-inhibitory peptide, GPFN, was identified from ultrafine *L. barbarum* pomace powder using enzyme assays and in silico analysis [20]. A different study used trivalent iron salts to release sweet-enhancing volatiles from pomace [21], while supercritical carbon dioxide extraction recovered an oil rich in zeaxanthin dipalmitate from a seed–peel residue [22]. The peptide-containing powder, treated pomace, and recovered oil are distinct products of different processes. Fermentation changes residual biomass further, and feeding and multi-omics studies examine these altered residues rather than intact leaves or berries [32,54,55]. Figure 2 summarizes the principal compositional groups without implying equal abundance among materials.

## 5. Processing and Applications in Food Matrices

### 5.1. Teas and Fermented Products

Tea aroma varies with both plant line and processing. Green- and white-tea procedures produced different volatile profiles across 25 goji lines [6], while bud-versus-leaf comparisons showed variation in phenolics and enzyme inhibition responses [7]. A 2026 metabolomics study followed Ningqicai No. 1 shoot tips from fresh material through withering, fixation, stir-firing, and fragrance enhancement [56]. Early and heated stages differed in metabolite profiles, but the reported pre-injection β-glucosidase treatment complicates attribution of glycoside-to-aglycone changes to tea manufacture alone. The study supports stage-associated chemical variation more directly than its proposed enzymatic mechanism. Microbial processing offers a separate route: a 12-day pile fermentation study reported microbial succession accompanied by changes in volatiles, bitterness, and astringency [8]. Only its abstract was available for appraisal. Together, the studies indicate that genotype, tissue maturity, thermal treatment, and fermentation contribute different sources of flavor variation.

### 5.2. Extraction and Fractionation

Aqueous extraction followed by ultrafiltration and nanofiltration clarified and concentrated leaf constituents [57]. Membrane fractionation separates components from an extract mixture, whereas solvent-assisted extraction changes their initial recovery from the plant matrix. This difference is important when comparing an aqueous concentrate with the phenolic-enriched fractions obtained using eutectic solvents.

Three recent studies used choline chloride–lactic acid, betaine–ethylene glycol, and betaine–1,3-butanediol–lactic acid, respectively, to extract leaf phenolics or flavonoids [58,59,60]. Downstream separation is important to interpreting their products. In the betaine–ethylene glycol study, the extract underwent D101 resin separation, washing with 3.5 bed volumes of water, elution with 70% ethanol, and lyophilization [59]. The betaine–1,3-butanediol–lactic acid study also used D101 resin but reported a two-bed-volume water wash and 75% ethanol elution before solvent removal and freeze-drying [60]. The choline chloride–lactic acid paper was available only at abstract level, so its downstream cleanup could not be appraised here [58].

The betaine–ethylene glycol study reported extraction performance above 90% after three solvent-reuse cycles [59]. This is a relative extraction result, not a measurement of solvent mass recovery or residual solvent in the final ingredient. Likewise, the reported biological assays on purified fractions do not assess dietary safety of the original extraction mixtures [59,60]. Resin washing and ethanol elution establish that a cleanup step was performed, but do not by themselves quantify residual exposure. Food-use interpretation, therefore, depends on the final ingredient rather than on the starting extract: residual concentrations and the amount consumed are needed to relate purification to exposure. Neither extraction yield nor the designation “natural” establishes food suitability or environmental superiority.

Ion-exchange separation of caffeoyl spermidine derivatives from *L. barbarum* leaf tea produced a combined fraction with reported recovery of 92.5% and purity of 87.6% [44]. Further preparative separation yielded individual derivatives. The result demonstrates enrichment of a compound class present in tea, but the concentrated fraction is chemically different from the beverage. Its production also introduces salt and ethanol recovery operations, for which the study does not establish a complete manufacturing mass balance or commercial cost.

### 5.3. Cereal and Bakery Matrices

Leaf polysaccharides modified wheat dough at additions of 0.5–2.0% of flour weight [9,49]. In the 2021 experiment, the degraded preparation increased extension resistance and extension area under selected addition levels, with concurrent changes in protein interaction and microstructural measurements [49]. The 2024 study found dose-dependent changes in hydration, texture, extensographic behavior, and cooking loss, but not a significant improvement in the trained-panel sensory scores of rinsed dough [9]. Thus, an instrumental rheological change did not translate automatically into a sensory advantage. The 2024 results also describe the color of baked dough, but the reported methods provide no corresponding baking protocol. Without those conditions, the color observations cannot be related to a reproducible finished-product test. Within-study dose comparisons remain more informative than a combined molecular-weight–response relationship because the preparations are not demonstrably equivalent.

In muffins and cookies, a berry-processing by-product increased protein, fiber, and phenolic measures while also changing physical and sensory properties [19]. The publisher abstract describes product-dependent effects but does not support a detailed comparison of acceptable substitution limits. This multi-component addition differs from the purified leaf polysaccharides used in dough experiments: its fiber and other residual components remain in the baked product alongside extractable phenolics.

### 5.4. Delivery Systems, Emulsions, and Savory Foods

Protein-based carriers alter the stability of leaf constituents and co-encapsulated carotenoids. An abstract-level report describes whey protein isolate and bovine serum albumin nanoparticles containing a leaf extract [10]. In a full-text study, purified *L. barbarum* leaf flavonoids were coupled to whey protein using hydrogen peroxide and ascorbic acid, followed by dialysis [11]. The conjugates improved β-carotene retention during storage and simulated digestion relative to whey protein alone. However, the model emulsions contained 0.02% sodium azide as a preservative and were prepared at pH 8—they were not edible test formulations. Their endpoint was retained β-carotene content, not micellar bioaccessibility or absorption. A separate abstract-level study used collagen–leaf-flavonoid conjugates for lutein emulsions [61]. Differences in protein, carotenoid, pH, and preparation prevent treating these systems as a common validated food ingredient.

Leaf-polyphenol liposomes were examined for storage stability, release into phosphate buffer at pH 7.4, and protection of hydrogen-peroxide-treated L929 fibroblasts [62]. Buffer release is not simulated gastrointestinal digestion. A separate leaf-polysaccharide Pickering emulsion study did include simulated digestion, but curcumin loading destabilized the tested emulsions [63]. Demineralization increased free-fatty-acid release while reducing micellar curcumin recovery. These divergent responses show why greater lipid digestion cannot be equated with greater bioaccessibility of the encapsulated compound. Neither experiment measured human absorption or the sensory performance of a finished food.

Savory-food experiments show a clearer trade-off between ingredient addition and taste. The abstract of a chicken soup study reports changes in composition, volatiles, and sensory attributes after addition of bud tea [12]. In a full-text stewed chicken study, *L. barbarum* bud tea was added at 15, 20, or 25 g/kg [64]. The 20 g/kg treatment achieved the highest tenderness and overall sensory scores among the tested formulations, whereas 25 g/kg increased bitterness. These scores came from ten trained assessors, not a consumer sample. The response consequently identifies a formulation-specific balance rather than a generally optimal intake.

A 70% ethanol leaf-flavonoid extract was evaluated at 0.01%, 0.1%, and 1% in bowl-steamed Tan lamb [65]. After seven days of storage and reheating, the 0.1% treatment most consistently limited warmed-over flavor and associated lipid oxidation volatiles. The ten-person trained-panel experiment thus favored an intermediate rather than maximal addition. The methods specify −4 °C storage, whereas the results describe 4 °C, leaving the storage regime unresolved and preventing an inference about refrigerated shelf life. The within-experiment flavor comparison remains relevant, but the lamb and chicken studies used different matrices, preparations, and sensory protocols and do not establish a common optimal inclusion level.

Table 2 compares the tested preparations, experimental systems, and measured outcomes in processing and food application studies.

## 6. Digestion and Biological Activities

### 6.1. Digestion and Bioaccessibility

Digestion changes the fraction available to subsequent assays. A leaf-phenolic study combined simulated gastrointestinal digestion with an alcohol-damaged AML-12 hepatocyte model and observed activity of the post-digestion fraction within that cell system [66]. The choline chloride–lactic acid study, available here at abstract level, reported losses of recoverable phenolics and assay responses during simulated digestion [58]. Conversely, whey-protein–leaf-flavonoid emulsions retained more β-carotene than the protein-only comparator [11]. Chemical recovery after digestion and release into a potentially absorbable fraction are different endpoints: retention alone does not demonstrate bioaccessibility, and neither measures intestinal absorption or systemic bioavailability. Sequentially extracted leaf polysaccharides also differed in in vitro fermentation behavior, extending the evidence to a colonic model rather than solely the upper-gastrointestinal compartment [47].

### 6.2. Biological Models and Interpretation

The biological literature examines different preparations and endpoints. Wild and cultivated *L. barbarum* leaf extracts differed in phenolic profiles and in chemical or enzyme-based assay responses [39]. Flavonoid preparations were associated with changes in metabolic, oxidative stress, and microbiota endpoints in high-fat-diet-fed mice, and with redox-related responses in cells and *Caenorhabditis elegans* [67,68]. Polysaccharide studies instead include splenocyte proliferation and an ovalbumin-induced asthma mouse model [46,69]. These findings cannot be collapsed into a single mechanism: the interventions differ in composition, concentration, and disease model. In particular, an antioxidant assay or a change in microbial abundance does not establish the pathway by which a leaf-containing food would affect human health.

Target-animal feeding studies have a different interpretation from rodent disease models. Branch roughage in dairy cows, mixed branch–leaf biomass in sheep, and processing residue in grass carp were tested for outcomes relevant to those feed uses [29,30,70]. Their dietary formulations and endpoints are directly relevant to the target species but do not provide human food efficacy evidence.

**Figure 3:**
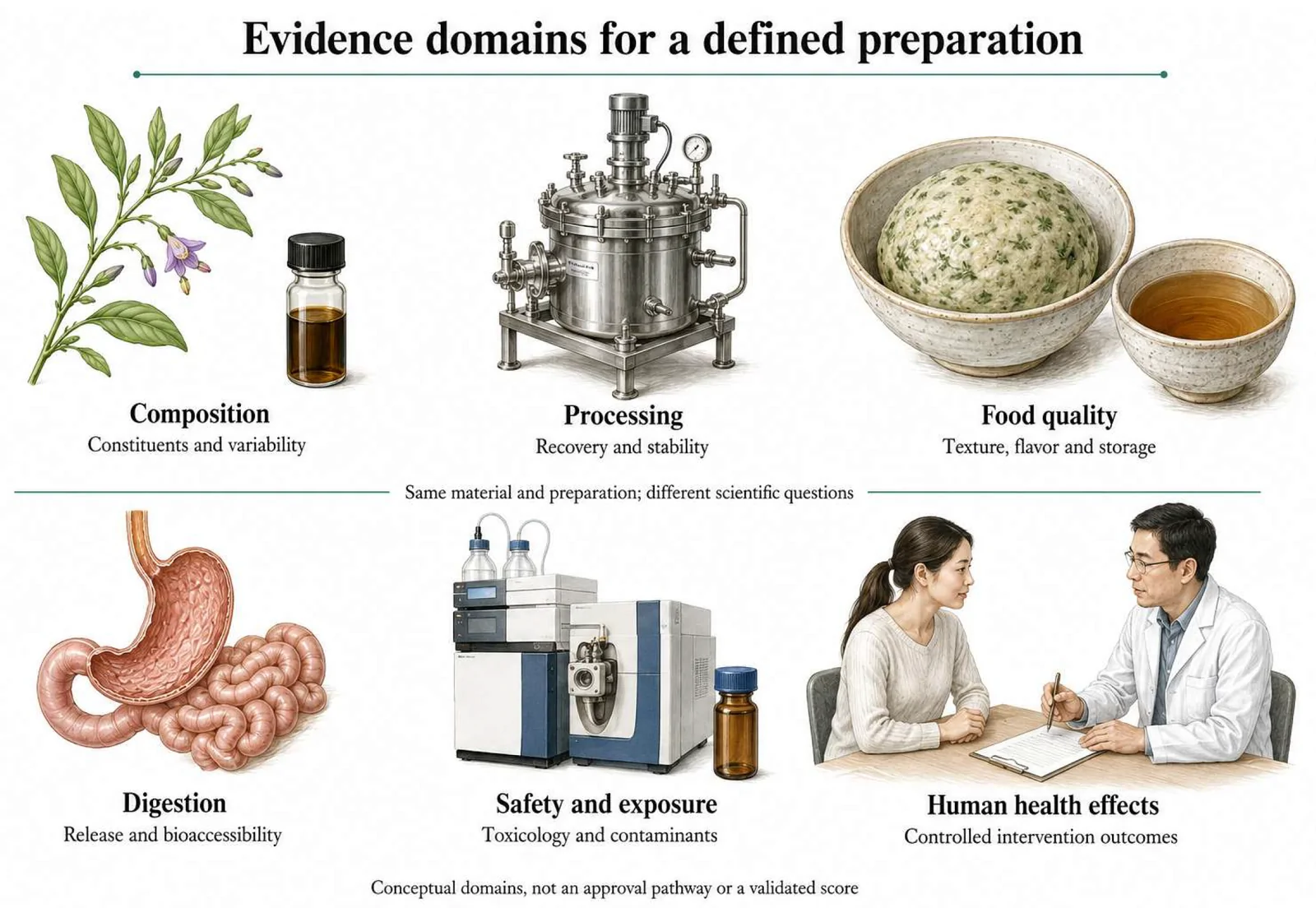
Different claims require different evidence. Composition, processing performance, food quality, digestion, safety, and human health effects are separate evidence domains linked to a defined preparation. The layout is non-sequential: it is not a six-stage approval pathway, and not every type of claim requires all domains. Illustrations are AI-assisted schematic artwork.

Clinical efficacy of a defined leaf- or bud-derived food is not established by the studies represented in this review. Their principal outcomes are chemical, technological, simulated-digestion, cell, or animal measurements. Figure 3 distinguishes these evidence domains and the different outcomes they address.

## 7. Safety, Quality Control, and Regulatory Considerations

### 7.1. Preparation-Specific Safety

A toxicology study examined roasted *L. chinense* leaves extracted with 30% ethanol, concentrated and spray-dried, and standardized to a kaempferol glycoside [71]. Acute toxicity, 14-day repeated-dose exposure, and genotoxicity assays did not identify evident toxicity under the tested conditions. The exposure duration limits this result to short-term safety of the specified extract. Longer-term intake, fresh-leaf consumption, and preparations from other species were not evaluated in that experiment.

In a direct organ comparison, solasonine and 5,6-dihydrosolasonine were detected in the tested fruit powder but not in the analyzed leaf or root-bark samples [23]. Non-detection is conditional on the sampled material and analytical method—it does not establish absence from all production batches. The cited evidence also does not resolve product-specific allergenicity or a broad antinutrient profile. These uncertainties are particularly relevant when extraction concentrates constituents or when a whole-leaf food replaces an infusion.

### 7.2. Pesticides, Elements, and Biogenic Amines

Pesticide occurrence in dry tea and transfer into the beverage are related but distinct observations. A field study found that spray adjuvants affected deposition differently on leaves and fruits [72]. In market surveillance, Li et al. detected 23 pesticides in 28 of 30 goji bud-tea samples [13]. The main-text description of these market samples did not assign a Latin species, and the findings, therefore, remain product-specific rather than species-specific. Their infusion experiment used one selected sample, 2.0 g tea per 100 mL boiling water, and three successive brews. Transfer varied by compound, increased with infusion duration in the tested conditions, and generally declined over successive brews. Although the modeled dietary risks remained below the authors’ thresholds, the result depends on that exposure model and cannot substitute for batch compliance testing. A one-sample transfer experiment also leaves uncertainty about seasonal and manufacturing variation.

Processing effects on residues have also been measured in goji fruit. Drying concentrated the four investigated pesticides, whereas decoction and brewing reduced transfer into consumed products under the tested conditions [73]. The different organs and processing protocols preclude using these numerical factors to estimate bud-tea exposure. The elemental analysis report available here at abstract level supports occurrence in *L. barbarum* leaves, not a serving-based exposure or compliance assessment [35]. The biogenic-amine study sampled eight *L. barbarum* materials: young and mature leaves, young and mature stems, main-stem bark, flowers, fruit, and roots [53]. The leaf–bark comparison discussed here is a subset of that design, and the sampled bark was main-stem bark rather than medicinal root bark. Histamine was higher in the tested main-stem bark, whereas the tissue patterns of other amines differed. These measurements neither establish a common hazard for all biogenic amines nor quantify the dose consumed in a leaf infusion.

### 7.3. Identity, Specification, and Regulatory Status

Published leaf position and harvest period studies show that a chemically variable raw material can retain the same commercial name [37,38]. A fingerprint can distinguish such batches, but no single phenolic marker describes aroma, microbial quality, contaminant burden, and technological function simultaneously. Whole-leaf powder also differs from tea because the insoluble material is consumed rather than discarded. These distinctions explain why a specification established for a dry leaf extract cannot be applied unchanged to the beverage.

Processing creates further differences in the attributes that define an ingredient. Molecular-size distribution and degradation history distinguish the polysaccharides used in dough experiments [9,48,49]. Seed and peel proportions distinguish the oil-recovery residue from other pomaces [22], whereas fiber content and residual foliage are central to branch and mixed branch–leaf feeds [29,30]. Quality control is, therefore, preparation-specific, not a common phenolic threshold for every goji-derived resource.

The regulatory examples considered here concern China and the European Union, not a global assessment. In the European Union, the Novel Food Status Catalogue is non-binding and non-exhaustive. A history of significant consumption before 15 May 1997, the identity of the preparation, and the applicable consultation or authorization route are relevant to novel-food status [74,75]. Historical consumption of one plant part does not by itself establish the status of a concentrated extract or another organ. The Commission also identifies extraction solvents used in food production as a separate regulatory subject under Directive 2009/32/EC [75]. Novel-food status and extraction solvent compliance are thus different questions, and the cited guidance does not establish authorization of the specific eutectic mixtures discussed above. A regulatory status record is also distinct from toxicological evidence.

In China, GB/T 18672-2014 addresses wolfberry fruit, rather than establishing a specification for leaf tea or concentrated leaf ingredients [76]. A Tibet Autonomous Region notice records an enterprise standard filing for goji bud tea, while expressly stating that filing is not evidence of product safety or a prerequisite for production licensing [77]. The notice thus documents a filing, not nationwide authorization or a safety endorsement. Product development requires confirmation of the applicable current rules for the precise species, part, process, and market.

## 8. Side-Stream Valorization: Tested Routes and Constraints

### 8.1. Berry-Processing Residues in Food and Feed

Circular valorization refers here to recovery or reuse of residual material from an existing production chain. It does not mean that every recovered product has demonstrated economic or environmental superiority. Goji by-product incorporation in muffins and cookies retains multiple components in a food matrix [19], whereas peptide recovery, iron-assisted volatile release, and residue-oil extraction isolate or modify selected fractions [20,21,22]. These routes differ in product yield, additional reagents, and the remaining biomass, and their results are not directly comparable measures of value.

A sequence of oil recovery, extraction of other constituents, and use of the remaining solids is a possible cascade, but it has not been demonstrated as an integrated goji process by the studies considered here. The individual experiments do not provide a common-batch mass balance, techno-economic analysis, or life-cycle comparison of competing routes. Greater extraction yield can also involve additional solvent, water, drying, or separation. Consequently, “green” extraction and “high-value” recovery remain process claims requiring evidence beyond laboratory yield.

Raw and fermented residues have been incorporated into sheep, lamb, and grass-carp diets, with measurements of growth, immune, intestinal, or meat-quality endpoints [32,54,55,70]. Other sheep studies measured energy and nitrogen metabolism, methane emissions, rumen fermentation, and digestive or antioxidant indices [78,79]. Silage experiments examined fermentation quality and microbial communities after addition of goji by-products [80]. These studies address different feed-processing questions, and a fermentation-associated change in residue composition is not necessarily equivalent to a benefit from the untreated residue.

Interpretation depends on the basal diet and inclusion level as well as on residue composition. Replacing part of a conventional feed changes both the added material and the displaced ingredient, while fermentation can alter moisture, nutrient availability, and storage behavior. The reported animal or silage outcomes, therefore, apply to the tested formulations. They do not establish that the same residue can enter a human food chain or that a feed route is economically preferable to another recovery option.

### 8.2. Orchard Branches and Non-Food Leaf Biomass

Woody branches have been examined as dairy cow roughage and as a cultivation substrate [29,31]. These applications exploit structural biomass rather than the edible-leaf composition emphasized in food studies. Separately, leaf biomass has undergone enzymatic saccharification with fungal enzyme cocktails [81]. This is a leaf-derived biorefinery experiment, not a branch conversion study, and its carbohydrate-release endpoint is distinct from nutritional or sensory performance. Table 3 separates the materials and the direct evidence for each use.

Food incorporation and ingredient recovery favor different properties of the same biomass. Retaining a multi-component residue in bakery products can increase fiber and phenolic measures but also alters texture [19]. Fractionation yields more concentrated constituents, at the cost of additional separation and recovery operations [20,21,22]. Feed and cultivation routes instead use larger residual fractions with fewer requirements for isolation. Comparisons of their net value remain limited by the absence of common-input mass balances, costs, and environmental measurements.

**Figure 4:**
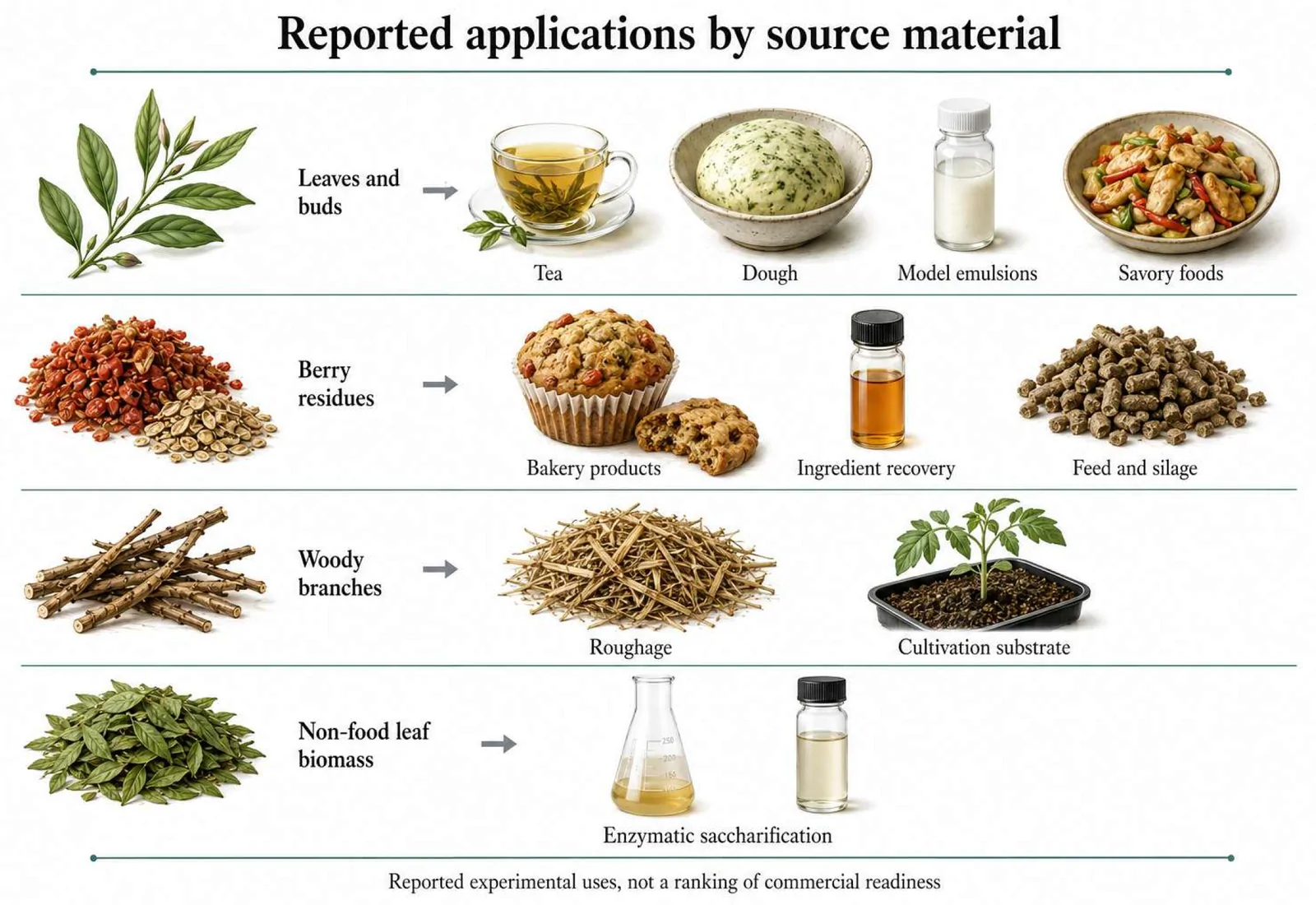
Reported applications of distinct goji resources. The unranked map links leaves and buds to food and formulation studies, berry residues to food/feed or recovery experiments, and woody branches to agricultural uses. Non-food leaf saccharification is identified separately. Layout does not denote readiness, safety approval, or superiority. Illustrations are AI-assisted schematic artwork.

Reported applications consequently fall into distinct material and product groups (Figure 4). Leaf and bud experiments chiefly examine tea chemistry and formulated food performance, berry residues enter food incorporation, fraction recovery, and feeding trials, and woody branches are used as structural agricultural biomass. This distribution reflects the systems studied, rather than a demonstrated order of commercial readiness.

## 9. Conclusions

Goji leaves and buds are compositionally variable food materials, with differences attributable to species, cultivar, tissue maturity, harvest, and processing. Research now includes tea, dough, colloidal carriers, and savory foods, but the outcomes are not uniformly favorable: improved instrumental properties or greater phenolic content can coexist with bitterness, altered texture, or unchanged sensory scores. The most informative results are, therefore, the preparation- and dose-specific comparisons, rather than a general claim of leaf bioactivity.

Simulated digestion and biological models provide preparation-specific observations, while safety information remains limited to particular extracts, batches, and exposure models. The main food development uncertainties concern reproducibility of composition and technological performance, serving-based exposure, and the balance between enrichment and sensory quality. Berry-processing residues and woody branches have separate recovery and agricultural uses. Their comparative environmental and economic performance remains unresolved because integrated, common-input evaluations are scarce.

## Figures and Tables

**Table 1:** Illustrative within-study comparisons of goji leaf composition from selected full texts.

Species and Material	Origin or Comparison	Preparation and Analytical Basis	Selected Reported Value	Interpretation
*L. barbarum* cultivated leaves	Four cultivated varieties supplied by a Ningxia institute	Young pre-flowering leaves; 50 °C vacuum drying; ether pretreatment; HPLC; dry-mass basis	Rutin, 16.03–16.33 mg/g [36]	Population range under one method; not a universal specification
*L. barbarum* wild leaves	Wild populations: Qinghai, Xinjiang, Ningxia, Inner Mongolia	Young pre-flowering leaves; 50 °C vacuum drying; ether pretreatment; HPLC; dry-mass basis	Rutin, 6.24–7.55 mg/g [36]	Lower than the cultivated populations in this study only
*L. barbarum* leaves	Romanian cultivars Biglifeberry and Erma and one wild population	Shade-dried; 70% methanol; ultrasound; LC-DAD; dry-mass basis	Chlorogenic acid, 17.811, 24.887, and 0.511 mg/g, respectively [39]	Demonstrates a large cultivar/origin contrast under a common method
*L. barbarum* fruitless cultivar leaves	Ten harvests, Ningxia	Dried leaf; 90% methanol; ultrasound; HPLC-DAD; dry-mass basis	Rutin, 0.66–16.96 mg/g; chlorogenic acid, 0.72–15.31 mg/g [38]	Harvest date changed both markers by more than an order of magnitude
*L. barbarum* fruitless cultivar leaves	Same ten-harvest series	Same method	Catechin, 2.63–16.33 mg/g; epicatechin, 1.50–20.90 mg/g [38]	A single batch value is insufficient without harvest definition
*L. barbarum* tip-bud leaves	Ningqi No. 1, Ningxia	70% ethanol; ultrasound; HPLC-MS/MS with naringenin-equivalent semiquantitation	Chlorogenic acid, 2105.05 ± 77.25 μg NE/g [37]	Semiquantitative basis prevents direct comparison with external-standard HPLC values
*L. barbarum* old basal leaves	Ningqi No. 1, Ningxia	Reflux, ultrasound, or cellulase extraction; same semiquantitative method	Chlorogenic acid, 412.51–1479.99 μg NE/g [37]	Extraction alone changed the apparent value by more than threefold
*L. barbarum* commercial leaf powder	Direct fruit–leaf–root-bark comparison, Ningxia	70% ethanol; ultrasound; UPLC-QTRAP-MS/MS; dry-powder basis	Chlorogenic acid, 1577 ± 4 μg/g; rutin, 663 ± 15 μg/g [23]	Directly comparable only within this organ-comparison experiment

Note: This is a selected, non-exhaustive set of within-study contrasts, not a representative concentration distribution. Values retain the original dry-mass and calibration basis. NE values are naringenin-equivalent semiquantitation and are not directly comparable with compound-specific calibration. DAD, diode-array detection; HPLC, high-performance liquid chromatography; LC, liquid chromatography; MS/MS, tandem mass spectrometry; NE, naringenin equivalents; QTRAP, quadrupole linear ion trap; UPLC, ultra-performance liquid chromatography. Source locations, replication as reported, and unit conversions are provided in Supplementary Table S3.

**Table 2:** Experimental systems and reported outcomes in processing and food application studies.

Material and Source Access	Experimental System	Measured Result	Interpretive Limit
*L. barbarum* leaf tea [6] (F)	Green/white processing across 25 lines	Line and process differences in volatile profiles and aroma	Aroma profiling is not population-level consumer acceptance.
*L. barbarum* shoot-tip tea [56] (F)	Five processing stages; metabolomics	Stage-associated differences in metabolite profiles	Pre-injection enzyme treatment complicates native glycoside interpretation; manufacturing conditions need clarification.
Goji bud tea [8] (A); species unassigned	12-day pile fermentation	Reported microbial and flavor profile changes	Process detail and microbial safety not appraised from the abstract.
Bud tea [12] (A), species unassigned; *L. barbarum* bud tea [64] (F)	Chicken soup; stewed chicken at 15, 20, 25 g/kg	Composition and sensory changes; 20 g/kg gave highest tested overall score in stewed chicken	Soup and chicken are separate formulations; chicken panel comprised ten trained assessors.
*L. barbarum* leaf polysaccharides [9,49] (F)	Wheat dough; additions 0.5–2.0% of flour weight	Dose-dependent hydration, texture, and extensographic changes	Baked-dough color described in [9] without a corresponding baking protocol; preparations differ, so no pooled molecular-weight response.
Berry-processing by-product [19] (A); species unassigned	Muffins and cookies	Reported nutrient/phenolic increases and physical/sensory changes	Detailed inclusion limits not verified in full text.
*L. barbarum* leaf flavonoids [11] (F)	Whey protein conjugates; β-carotene emulsions at pH 8	Improved carotenoid retention during storage and simulated digestion	Contains 0.02% sodium azide; not an edible test formulation. Retention is not bioaccessibility.
*L. barbarum* leaf extract/flavonoids [10,61] (A); *L. barbarum* leaf polysaccharides [63] (F)	Protein nanoparticles and lutein/curcumin model emulsions	Carrier-dependent outcomes; curcumin loading destabilized the tested Pickering emulsions	Different formulations; demineralization increased lipid digestion but reduced micellar curcumin recovery. Abstract-only details not appraised.
*L. barbarum* aqueous leaf extract [57] (F)	Ultrafiltration and nanofiltration	Clarification and concentration of extract fractions	Not a finished-food test or commercial mass balance.
*L. barbarum* leaf extracts [58] (A), [59,60] (F)	Ultrasound/eutectic extraction; D101 resin, water washing and ethanol elution in the two full-text studies	Extraction and purified fraction assays; solvent-reuse extraction performance in [59]	Cleanup is not a final-residual measurement. Purified fraction bioactivity does not establish solvent mixture safety.
*L. barbarum* leaf tea [44] (F)	Ion exchange and preparative separation	Combined caffeoyl spermidine fraction: 92.5% recovery, 87.6% purity	Enriched ingredient, not beverage; manufacturing cost and full solvent balance unresolved.
*L. barbarum* leaf-flavonoid extract [65] (F)	Steamed Tan lamb, 0.01–1%; seven-day storage and reheating	0.1% most consistently limited warmed-over flavor	Ten trained assessors; methods −4 °C versus results 4 °C. No refrigerated shelf-life inference.
*L. barbarum* pomace [21] (F); *L. barbarum* seed–peel residue [22] (F)	Iron-assisted volatile release; oil extraction/microencapsulation	Release of volatiles; recovery of carotenoid-rich oil	Different parent fractions; no common mass balance or route comparison.

Note: F, full text available for appraisal; A, abstract-level source. Source access is not a risk-of-bias rating. Detailed extraction and unresolved checks are identified in Supplementary Table S2.

**Table 3:** Preparation-specific safety, exposure, regulatory, and side-stream evidence.

Preparation or Material	Evidence and Source Access	Supported Observation	Unresolved Applicability
Roasted *L. chinense* leaf extract	Acute, 14-day repeated-dose and genotoxicity tests [71] (F)	No evident toxicity in the tested conditions	Not chronic safety; not equivalent to other species, teas, or concentrated fractions.
Commercial *L. barbarum* leaf powder	Targeted fruit/leaf/root-bark comparison [23] (F)	Two targeted glycoalkaloids not detected in tested leaf samples	Batch- and method-specific non-detection, not a population survey.
Market goji bud tea; Latin species unassigned in tested samples	Occurrence in 30 samples; one-sample infusion transfer [13] (F)	Residues and compound-dependent transfer; modeled risk below study thresholds	Transfer variability and other consumption scenarios remain unresolved.
*L. barbarum* leaf elements, eight-part amine comparison, and field pesticide deposition	Elements [35] (A), biogenic amines [53] (F), pesticide deposition [72] (F)	Analytical or field observations for the tested materials	Amine sampling: young/mature leaves, young/mature stems, main-stem bark, flowers, fruit, roots. Occurrence is not serving-based exposure; elemental source is abstract-only.
Leaf/bud products and extracts; species depends on the product and document	EU framework and Chinese fruit standard/bud-tea filing [74,75,76,77] (O)	Different legal instruments and product identities	Historical use, filing, authorization, and demonstrated safety are not equivalent.
Berry by-product [19], species unassigned; *L. barbarum* pomace/seed–peel fractions [20,21,22]	Bakery [19] (A); peptide, flavor, and oil experiments [20,21,22] (F)	Food incorporation or ingredient recovery in separate processes	No integrated cascade, common-batch LCA, or techno-economic comparison.
*L. barbarum* raw or fermented berry residue	Sheep, grass-carp, and silage studies [32,70,80] (F)	Formulation- and target-use-specific outcomes	Not evidence for human efficacy or automatic transfer to a human food chain.
*L. barbarum* woody branches; mixed branches and leaves	Cow roughage, sheep diet, and substrate studies [29,30,31] (F)	Tested agricultural uses of defined biomass	Wood and mixed foliage are not interchangeable edible-leaf ingredients.
*L. barbarum* non-food leaf biomass	Enzymatic saccharification [81] (A)	Reported carbohydrate release in a biorefinery process	Not a branch conversion experiment or an edible-leaf study.

Note: Rows address different questions and are not ordered stages. F, full text available for appraisal; A, abstract-level source; O, official document. Source-access labels are not quality ratings.

## Data Availability

No new data were created or analyzed in this study. Data sharing is not applicable to this article.

## Supplementary Materials

The following supporting information can be downloaded at: https://www.mdpi.com/article/10.3390/foods15183298/s1. Supplementary Methods S1: Archived search procedures, revision-update log, source-access definitions, and evidence description. Supplementary Table S1: Scope of related reviews. Supplementary Table S2: Source register. Supplementary Table S3: Quantitative composition extraction. Supplementary Data S1a: Archived discovery ledger (29 July 2026). Supplementary Data S1b: Revision-addition records (6 September 2026). Supplementary Data S2a: Reference number crosswalk. Supplementary Data S2b: Current manuscript citation audit.
