# Supplementary material for: Goji Leaves and Buds as Food Resources: Composition, Processing, Safety, and Links to Side-Stream Valorization
Source: Foods. 2026 Sep 17;15(18):3298. doi: 10.3390/foods15183298 (PMC13606126; doi:10.3390/foods15183298)
Supplement: Supplementary file 1 [file foods-15-03298-s001.zip › Supplementary_Methods_S1.pdf]

Supplementary Methods S1

Archived search procedures, revision-update log, source-access definitions, and evidence description.

Review design and coverage

This is a critical narrative review of leaves and buds as food resources, with selected berry-processing and orchard-biomass comparisons. It is not a prospectively registered systematic review or meta-analysis. PubMed and Europe PMC form the archived bibliographic search layer. OpenAlex, Crossref, publisher discovery and citation checking supplied additional records. Web of Science, Scopus, FSTA and CAB Abstracts were not searched. This limits coverage of food technology, agronomy and industrial side streams.

Dates and record accounting

The retained PubMed and Europe PMC update is dated 29 July 2026 and returned 575 and 2160 records, respectively (2735 provider records). OpenAlex and Crossref exports contained 1518 and 1200 records; 240 supplementary-discovery records passed the material-specific prefilter. An earlier OpenAlex snapshot was reused after a rate-limit error, so its records do not represent complete coverage on 29 July. The title-grouped ledger contains 2247 records: 1246 core candidates, 742 context candidates, 242 low-priority labels and 17 manual-check flags. These automated labels are not completed eligibility decisions, unique-study counts or a PRISMA flow.

Revision update

On 6 September 2026, targeted checks added a Cheongsoon cultivar study (10.9787/KJBS.2025.57.4.483) and a tea-processing metabolomics study (10.7717/peerj.21226). Their publisher/full-text records are logged separately in Data S1b. This was not a repeat search of all databases. The two additions are not combined with the archived discovery counts in Data S1a. Retrieval of full text for an already cited paper changes its access record, not its status as a newly discovered citation.

Claim-bounded follow-up

On 7 September 2026, the 41 full-text entries previously carrying generic re-extraction notes were checked against the source sections relevant to their use in this review. Table S2 records each check and its interpretive boundary. Cultivation and branch-substrate species assignments were corrected; species-unassigned commercial tea samples and abstract-only records remain explicitly unassigned. These checks do not constitute a new database search, independent duplicate extraction, or validation of every finding in each paper. Source-access counts remain 66 full texts, 10 abstract-level records and five official documents.

Query implementation

The archived Python search script used the exact query strings reproduced below. PubMed and Europe PMC used title/abstract organism and material searches. The 12 OpenAlex/Crossref discovery queries added food and use concepts; those service requests used a 1900-01-01 to 2026-07-29 publication-date filter. These sources and dates should not be represented as equivalent database coverage. The script was project-specific Python code, not Zotero or EndNote deduplication; its historical Python interpreter version was not retained. A proof-stage consistency check was rerun with Python 3.12.14 on 15 September 2026 using the archived title and DOI normalization rules and the 0.85 title-similarity threshold. This check assessed the archived ledger and current source register; it did not constitute a new database search or external verification of all bibliographic records. The archived discovery ledger and its counts were not changed.

Title grouping and DOI reconciliation

The script normalizes titles with Unicode NFKD, lowercasing, replacement of non-ASCII-alphanumeric sequences by spaces, and whitespace collapse. DOI strings are lowercased and stripped of DOI URL prefixes and trailing punctuation. Records are grouped by identical normalized title. If titles attached to a DOI have a minimum pairwise difflib.SequenceMatcher ratio below 0.85, the DOI is flagged and suppressed rather than used to merge conflicting records. A DOI is retained for a title group only when one consistent DOI remains, except for a separately recorded manual title-DOI override. Source priority is PubMed, Europe PMC, OpenAlex, then Crossref. All formal-source records remain in the archive regardless of automatic relevance labels. The earlier PDF collection lacks a contemporaneous exclusion log and is not treated as a completed screening cohort.

Selection and source-access description

Cited primary studies concern a defined leaf/bud preparation or a specified comparator or side stream. Reviews supply context; official sources support document-specific regulatory statements. Table S2 separates primary research, review/contextual sources and official documents. F denotes a full text available for appraisal, A denotes abstract-level or bibliographic context in the retained revision record, and O denotes an official document. Access is not a quality rating. A source whose DOI appears only in another article reference list is not considered a full-text match.

Extraction and unresolved fields

The source register records material, species, preparation, experimental system and endpoint. A field not extracted in an earlier register is not labeled as absent from the source. Contextual and official sources may have no single species or preparation to which such fields apply. Revision notes distinguish selected direct checks from remaining extraction needs. Automated/AI-assisted checks do not constitute an independent human review, and no dual-screening or inter-rater procedure is claimed.

Quantitative comparisons

Table S3 contains 22 quantitative extraction rows checked against five full texts. The numerical values are unchanged; collection, sample-preparation and total-flavonoid calibration metadata for Dong et al. (2009) were corrected from the source. The comparative HPLC-table extraction aliquot is not separately specified in that paper and remains a reporting limitation. Current reference numbers link the rows to this revision. Main Table 1 is illustrative and non-exhaustive. No cross-study mean or molecular-weight response model is calculated. The 64.3 and 94.3 kDa values are reported separately because preparation and measurement comparability are not established, not because an original-paper error has been proven. Catechin 2.63-16.33 mg/g corresponds to about 6.2-fold, not more than tenfold.

Polysaccharide protocols: 2021 dough study

Al-Wraikat et al. (2021; main reference [49]; DOI 10.1016/j.lwt.2021.111372) used L. barbarum leaves collected in Ningxia in August 2019, dried at 60 °C for 24 h and milled through 40 mesh. Extraction used 50 g in 1000 mL water at 121 °C for 1 h. Sevage treatment, a resin named AD-8 in the source, and DEAE-52 chromatography preceded collection of the 0.1 M NaCl fraction. The reported degradation used 1 g polysaccharide in 100 mL water, 0.5 mL hydrogen peroxide and 0.88 g ascorbic acid (reported as a 1:1 ratio), followed by 2 h at room temperature, dialysis, 80% ethanol precipitation and freeze-drying. Table 1 reports 223.5 ± 1.13 and 64.3 ± 2.03 kDa. VISCOTEK laser-light-scattering gel chromatography is stated, but the description does not supply a complete calibration scheme. Dough testing used 0.5–2.0% additions; no baking procedure was located in the reported methods.

Polysaccharide protocols: 2022 characterization study

Al-Wraikat et al. (2022; main reference [48]; DOI 10.3390/polym14071404) used August 2020 leaves, with the same stated drying temperature and duration and 40-mesh milling. Extraction used 50 g in 1000 mL water at 120 °C for 1 h, followed by Sevage/protease treatment and selection of the DEAE 0.1 M NaCl fraction. The degradation protocol again describes 1 g/100 mL, 0.5 mL hydrogen peroxide and 0.88 g ascorbic acid for 2 h at room temperature. The 20, 40, 60 and 80 mM treatment series yielded reported molecular masses of 187.7, 144.9, 96.26 and 64.3 kDa, respectively. Section 2.11 describes gel-permeation chromatography with refractive-index and multi-angle light-scattering detectors, sodium nitrate eluent at 0.5 mL/min and 35 °C, and a 20 µL injection of a 1 mg/mL filtered sample. Molecular mass was estimated from refractive-index detection using dextran T calibration; radius of gyration was obtained from light scattering. These detector outputs are distinct.

Polysaccharide protocols: 2024 dough study

Al-Wraikat et al. (2024; main reference [9]; DOI 10.1016/j.lwt.2024.116753) describes extraction at 70 °C for 4 h twice, Sevage treatment and AB-8 resin purification; the corresponding DEAE 0.1 M NaCl fraction selection is not reported. Degradation is described for 100 mL solution using a 1:1 hydrogen peroxide/ascorbic-acid ratio at room temperature for 2 h, without absolute reagent amounts. The GPC procedure cites earlier methods without restating all detector and calibration conditions. The reported crude and degraded masses are 223.5 ± 1.13 and 94.3 kDa. Section 3.3.1

describes baked-dough color, but no corresponding baking procedure is provided in the methods. This limits reproducibility of that observation and does not prove that a baking experiment was absent.

Cross-study interpretation of polysaccharide size

Harvest batches, extraction temperature and duration, purification/fraction selection, degradation reporting and molecular-size measurement descriptions are not fully matched across the three papers. Thus, neither the degraded fractions nor their dough effects are pooled as a controlled molecular-mass series. The within-study degradation series in 2022 is retained as a condition-specific observation. The differences documented above do not prove why 64.3 and 94.3 kDa differ, and no published correction or author clarification is claimed. Both values are retained under their respective sources.

Source-specific interpretation

Different values across studies are distinguished from conflicting statements within one source. In the lamb study (10.1007/s11694-025-03549-3), storage is stated as −4 °C in methods and 4 °C in results; both reports are identified, and refrigerated shelf-life conclusions are not drawn. The 2026 tea-metabolomics paper includes beta-glucosidase pretreatment before injection, so stage-associated profiles are not interpreted as proof of native manufacturing-stage glycoside conversion. In the whey-protein emulsion study (10.1111/ijfs.16179), sodium azide in the experimental formulation and the carotenoid-retention endpoint distinguish model stabilization from edible-product validation or bioavailability. These limitations are specific to the affected claims and do not automatically exclude unrelated observations in each paper.

Material and endpoint corrections

The biogenic-amine study sampled eight materials: young and mature leaves, young and mature stems, main-stem bark, flowers, fruit and roots. The leaf/main-stem-bark comparison discussed in the review is a subset of this design; the bark is not medicinal root bark. Huang et al. (10.1016/j.indcrop.2023.117982) tested each of three tissues (bud, young and old leaves) by each of three extraction methods, giving nine combinations, rather than inseparably confounding tissue and extraction. The stem-fruit comparative study (10.1016/j.indcrop.2018.06.046) remains abstract-level and is not assigned a rhizome sample. The separate stem phenolic-amide paper used glioma stem-cell assays, not antimicrobial assays. Liposome buffer release and cell protection were separated from simulated digestion. The Pickering-emulsion study showed divergent effects of demineralization on free-fatty-acid release and micellar curcumin recovery. D101 resin washing and ethanol elution in the two full-text eutectic-solvent studies are recorded separately from solvent-reuse performance, mass recovery, final residual concentrations and mixture safety.

Evidence domains

Composition, process/formulation, food-matrix performance, simulated digestion/fermentation, biological models, toxicology/exposure, target-animal use and agricultural studies are described as different domains. No ordinal evidence level, development-stage score or application-readiness

ranking is assigned. A formal design-specific risk-of-bias assessment was not conducted. Results from different species, organs, formulations and intended uses are not combined into one validated product.

Human-intervention query limitation

The retained ClinicalTrials.gov check dated 29 July 2026 used the exact terms "Lycium barbarum leaf", "goji leaf", "wolfberry leaf" and "Lycium chinense leaf", with zero records recorded for those queries. The search did not systematically cover synonyms, buds, residues or all human studies. Consequently, the manuscript states that clinical efficacy is not established by the represented studies, not that no relevant human trial exists.

Supplementary file relationships

Methods S1 documents the archive and update. Table S2 contains 81 cited-source records; Table S3 contains quantitative extraction; Table S1 describes related reviews. Data S1a is the unchanged archived ledger and Data S1b lists the two revision additions. Data S2a maps editor-base and current reference numbers; Data S2b maps current citations to manuscript paragraphs and sections. These locations are not publisher PDF page/line numbers. Historical submission notes and ordinal-appraisal worksheets are not included.

PubMed query

(Lycium[Title/Abstract] OR goji[Title/Abstract] OR wolfberry[Title/Abstract] OR "Cortex Lycii"[Title/Abstract] OR "Lycii Radicis Cortex"[Title/Abstract]) AND (leaf[Title/Abstract] OR leaves[Title/Abstract] OR foliage[Title/Abstract] OR bud[Title/Abstract] OR buds[Title/Abstract] OR shoot[Title/Abstract] OR flower[Title/Abstract] OR flowers[Title/Abstract] OR stem[Title/Abstract] OR rhizome[Title/Abstract] OR "root bark"[Title/Abstract] OR "Cortex Lycii"[Title/Abstract] OR "Lycii Radicis Cortex"[Title/Abstract] OR branch[Title/Abstract] OR branches[Title/Abstract] OR pruning[Title/Abstract] OR pomace[Title/Abstract] OR residue[Title/Abstract] OR residues[Title/Abstract] OR seed[Title/Abstract] OR seeds[Title/Abstract] OR "seed residue"[Title/Abstract] OR "seed residues"[Title/Abstract] OR byproduct[Title/Abstract] OR by-products[Title/Abstract] OR "side stream"[Title/Abstract] OR "side-stream"[Title/Abstract] OR waste[Title/Abstract] OR peel[Title/Abstract] OR peels[Title/Abstract])

Europe PMC query

(TITLE_ABS:Lycium OR TITLE_ABS:goji OR TITLE_ABS:wolfberry OR TITLE_ABS:"Cortex Lycii" OR TITLE_ABS:"Lycii Radicis Cortex") AND (TITLE_ABS:leaf OR TITLE_ABS:leaves OR TITLE_ABS:foliage OR TITLE_ABS:bud OR TITLE_ABS:shoot OR TITLE_ABS:flower OR TITLE_ABS:stem OR TITLE_ABS:rhizome OR TITLE_ABS:"root bark" OR TITLE_ABS:"Cortex Lycii" OR TITLE_ABS:"Lycii Radicis Cortex" OR TITLE_ABS:branch OR TITLE_ABS:pruning OR TITLE_ABS:pomace OR TITLE_ABS:residue OR TITLE_ABS:seed OR TITLE_ABS:seeds OR TITLE_ABS:"seed residue" OR TITLE_ABS:"seed residues" OR TITLE_ABS:byproduct OR TITLE_ABS:"by-

product" OR TITLE_ABS:"side stream" OR TITLE_ABS:"side-stream" OR TITLE_ABS:waste OR TITLE_ABS:peel)

OpenAlex and Crossref discovery queries

Lycium leaves food composition

goji leaves tea processing aroma sensory

wolfberry leaves extraction phenolics polysaccharides

Lycium leaves nutrition minerals amino acids safety

Lycium leaves digestion bioaccessibility fermentation

Lycium leaf contaminants heavy metals pesticides alkaloids

Lycium flowers stems shoots phytochemistry food

goji branches pruning biomass feed

goji pomace residue byproduct food feed

goji seeds processing byproduct food

wolfberry processing side streams valorization

Lycii Radicis Cortex root bark safety
